# Predictors of CD4 count over time among HIV patients initiated ART in Felege Hiwot Referral Hospital, northwest Ethiopia: multilevel analysis

**DOI:** 10.1186/s13104-016-2182-4

**Published:** 2016-07-30

**Authors:** Lemma Derseh Gezie

**Affiliations:** Department of Epidemiology and Biostatistics, Institute of Public Health, College of Medicine and Health Sciences, University of Gondar, Gondar, Ethiopia

**Keywords:** Multilevel analysis, Linear mixed method, CD4 count, ART

## Abstract

**Background:**

The response of HIV patients to antiretroviral therapy could be measured by its strong predictor, the CD4+ T cell (CD4) count for the initiation of antiretroviral therapy and proper management of disease progress. However, in addition to HIV, there are other factors which can influence the CD4 cell count. Patient’s socio-economic, demographic, and behavioral variables, accessibility, duration of treatment etc., can be used to predict CD4 count.

**Methods:**

A retrospective cohort study was conducted to examine the predictors of CD4 count among ART users enrolled in the first 6 months of 2010 and followed upto mid 2014. The covariance components model was employed to determine the predictors of CD4 count over time.

**Results:**

A total of 1196 ART attendants were used to analyze their data descriptively. Eight hundred sixty-one of the attendants had two or more CD4 count measurements and were used in modeling their data using the linear mixed method. Thus, the mean rates of incensement of CD4 counts for patients with ambulatory/bedridden and working baseline functional status were 17.4 and 30.6 cells/mm^3^ per year, respectively. After adjusting for other variables, for each additional baseline CD4 count, the gain in CD4 count during treatment was 0.818 cells/mm^3^ (p value <0.001). Patient’s age and baseline functional status were also statistically significantly associated with CD4 count.

**Conclusion:**

In this study, higher baseline CD4 count, younger age, working functional status, and time in treatment contributed positively to the increment of the CD4 count. However, the observed increment at 4 year was unsatisfactory as the proportion of ART users who reached the normal range of CD4 count was very low. To see their long term treatment outcome, it requires further research with a sufficiently longer follow up data. In line with this, the local CD4 count for HIV negative persons should also be investigated for better comparison and proper disease management.

## Background

HIV/AIDS is one of the major public health problems in Sub-Saharan Africa, and Ethiopia, as one of these countries has been affected by the epidemic with a prevalence of 1.5 % [1]. Its burden has been high in the Amhara Region, including the catchment area of Felege Hiwot Referral Hospital where this study was conducted.

Currently, the only available treatment for HIV/AIDS is antiretroviral therapy (ART). Because HIV affects the CD4+ T-cell (CD4) counts in the human body, it can be employed to make appropriate decisions for the initiation of ART and proper management of the progression of the infection [2, 3]. For instance, it is recommended that after 3 months of treatment with ART, a patient should gain 50–100 CD4 cells/mm^3^ per year, and that an increment below this range could imply poor response to the treatment [4]. Patients’ CD4 count is also required to reach at least the lower limit of the CD4 count for the general healthy adult population (500 cells/mm^3^) [4] which otherwise can be an indication of immunologic failure.

However, in addition to HIV there are other factors which can affect CD4 cell counts. Among demographic variables, older ages are predictors of lower CD4 count response to ART [5, 6]. With regard to sex, females experienced better CD4 count response to ART compared to males as reported by a study done in North Ethiopia [7], but not according to other studies [5, 8].

Residence also has an effect on CD4 count at enrolment [9], and measures of accessibility, like distance of health facility from residence can have an effect on timing of diagnosis [10, 11] which in turn can affect the CD4 count at the initiation of ART [12]. Yet, patients with a deteriorated CD4 count at the initiation of ART due to late presentation would have poor response to ART [13].

While WHO clinical stage is reported to be an independent predictor of CD4 count at enrolment [9], it is not significantly associated with treatment response, according to a study that examined patients on ART for 12 months [7]. Similarly, studies reported that there is a positive association between baseline CD4 count and its size during treatment [14]; however, other studies also reported negative associations [6].

Available literature indicated that no study assessed the level of response to ART in the current and other similar study settings. On top of that, there were discrepancies in findings for some variables, like sex. Therefore, it is imperative that such a study which assesses the association between CD4 cell count against socio-demographic, time in treatment, accessibility, and clinical factors using the linear mixed method that enables to estimate the effect of variables considered by controlling for unmeasured variables.

Identifying factors which influence the level of CD4 count other than ART would help health professionals and patients to facilitate proper management and monitoring of health care interventions on HIV positive persons. Moreover, it helps to check whether HIV patients who initiated ART based on criteria formerly set (≤200 cells/mm^3^) were recovering to the normal range of CD4 count for the general healthy adult population which is 500 cells/mm^3^ or more [4] and is also equivalent to the cutoff point set recently to initiate ART for the adult HIV patients in the country [15]. Thus the current study was conducted with the objective of determining the level of CD4 count and factors associated with it after the initiation of ART at Felege Hiwot Referral Hospital, Ethiopia.

## Methods

### Study design and period

A retrospective cohort study was conducted to assess the predictors of CD4 count among adult ART users enrolled in the first 6 months of 2010 and followed up to mid 2014.

### Study area and population

The study was conducted at Felege Hiwot Referral Hospital located in the capital of the Amhara Regional State, Ethiopia. The hospital gives Voluntary Counseling and Testing services in a clinic to both physician and self-referred patients. The study population included HIV positive adults who initiated ART treatment in the referral hospital.

### Sample size and sampling procedure

All HIV positive adults who initiated ART in the hospital from January to June, 2010 (6 months) were included in the sample. Therefore, 1196 patients were selected for the sample, and 861 of them who had two or more CD4 count measurements were used in the multilevel analysis.

### Data collection procedures

The study exclusively used secondary data. Therefore, a data extraction check-list was designed and used to adopt the routinely collected data. A baseline CD4 count data were identified and collected from the registration documents of ART attendants which were collected from each patient at the initiation of ART. Subsequent CD4 count data were also taken from the same sources that were collected from each patient almost every 6 month. Similarly, other characteristics, like socio-demographic, time in treatment, accessibility, and clinical data were also collected from the registration documents of patients.

### Data structure, compilation and analysis strategy

Data were entered, cleaned, and analyzed using the statistical software called SPSS version 20. Some descriptive statistics were presented from all 1196 ART attendants using tables and graphs. However, only 861 patients who had two or more CD4 count measurements, including the baseline CD4 count data, were considered in the multilevel mixed model. That was because the CD4 count measurement just before the initiation of ART was considered as a covariate so that there should be at least one CD4 count reading that would be taken as a response variable after the initiation of ART.

The data structure had three levels of random grouping variables: the repeated measurements of CD4 count data (level-1), individual patients (level-2), and patients residence (level-3). With the identity link function, the multilevel linear mixed model (LMM) was employed to assess the effect of socio-demographic, time in treatment, accessibility, and other clinical variables on the repeated measure of CD4 count. By taking advantages of LMM’s flexibility and efficiency, both the random and fixed effects of predictors on CD4 counts were determined.

In this study, LMM was preferred to generalized estimation equation (GEE) to fit the data because the interest was mainly on the subject-specific (individual) interpretation of effects, not on the marginal (population) average effects. There was also interest of identifying variance sources and their components. Following this, the maximum likelihood (ML) parameter estimation method was employed. The overall effect of each explanatory variable on the CD4 count was tested by F-test, while the effect of each category of each factor was tested by t-test with the respective degrees of freedom.

To determine the model that best fits the data, exploratory data analysis was done first, especially by assessing individual CD4 count over time using individual trajectories. In addition to the descriptive individual trajectories, the unconditional null model was determined to further strengthen the relevance of the mixed model using the Intra-class correlation coefficient (ICC) calculated from it [16]. The null model would also serve as a baseline model for the purposes of comparison with later, more complex models. Then a model containing time as its fixed and random effect was determined before the final model containing the fixed effects of variables of interest, the random intercept, and the random slopes was fitted.

The model selection strategy used was comparing the Bayesian information criteria (BIC) of models considered. Accordingly, a model with a smaller value of BIC was selected as far as the change in BIC was statistically significant at Chi square with the change in the degree of freedom. The type of covariance structure and the magnitude of residual errors were also considered in model selection. In this regard, a model with the least within-individual variation when compared to other models residual variability was selected. In the present study, a significance level of 0.05 was taken as a cut point for all statistical tests.

## Results

### Patient characteristics and description of CD4 cell counts

A total of 1196 HIV positive adults who were attending ART at Felege Hiwot Referral Hospital were included in the study. The average age of study participants was 30.96 + 11.81 SD (standard deviation) and 666 (55.7 %) of them were females. The majority (721 or 60.3 %) of the patients were from Bahir Dar city where the hospital is located, while the remaining patients came from 36 other districts, the farthest being 265 km from the ART service center. These patients gave 6341 measurements of CD4 counts during the 4 years of retrospective follow up. The minimum and maximum numbers of CD4 count measurements per patient were 1 and 8, respectively, and the average number of measurements was 5.3.

Most of the participants (919 or 76.8 %) had baseline CD4 counts of less than 200 cells/mm^3^ which was the cutoff point to start ART for adults before the recent revision was made. Similarly, most participants had baseline WHO clinical stage of III or IV (955 or 79.9 %) and “working” functional status as of last observation (651 or 62.2 %). The average CD4 count increased from 150.27 (at baseline) to 453.13 (at 4 years); and in reference to baseline CD4 count, most (60.26 %) of the increment occurred during the first 6 months of follow up, while it was only 66.87 % at year four or an average increment of 6.61 % during the last three and half years (Table 1).

**Table 1:** Characteristics of ART attendants, Felege Hiwot Referral Hospital, 2010–2014

Characteristics	Number (percent)
Baseline age in year (n = 1196)	
15–20	149 (12.5)
21–30	473 (39.5)
31–40	388 (32.4)
41–50	128 (10.7)
≥51	58 (4.8)
Sex (n = 1196)	
Female	666 (55.7)
Male	530 (44.3)
Baseline functional status (n = 1047)	
Working	651 (62.2)
Ambulatory	345 (33.0)
Bedridden	51 (4.8)
Baseline WHO clinical stage (1196)	
Stage 1	61 (5.1)
Stage 2	180 (15.0)
Stage 3	809 (67.7)
Stage 4	146 (12.2)
First treatment class (n = 1196)	
AZT-3TC	732 (61.2)
D4t-3TC	36 (3.0)
D4t (30)	424 (35.5)
D4t (40) TDF-ddl	3 (0.3) 1 (0.08)
Baseline CD4 count (n = 1196)	
<200	919 (76.8)
200–349	218 (18.2)
350–499	32 (2.7)
≥500	27 (2.3)
Average CD4 count increment (compared to the baseline)^a^	
At 6 month (n = 861)	228 (60.26)
At 1 year (n = 787)	232 (60.66)
At 2 year (n = 701)	229 (60.33)
At 3 year (n = 569)	264 (63.73)
At 4 year (n = 378)	303 (66.87)

^a^The mean estimates were made under the condition of changing denominators because of attritions

Out of the 1196 participants who started ART, only 378 (31.6 %) had CD4 count measurements until the end of the follow up period, but only 142 (37.6 %) of these (378) had a CD4 count of at least 500 cells/mm^3^.

**Fig. 1:**
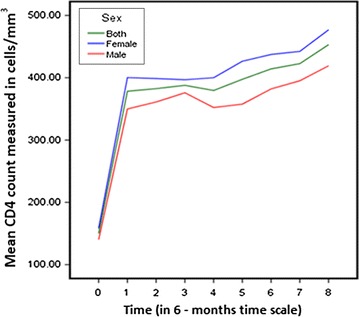
Trend of CD4 count by sex of ART attendants, Felege Hiwot Referral Hospital, 2010–2014

The difference between the average CD4 count of males and females increased gradually over time, males gaining a lesser average CD4 count. Both sexes had the highest average rates of increment (slope) of CD4 count during the first 6 months, and there after the rates of increment declined (Fig. 1).

### Exploratory data analysis

Because the rate of increment in CD4 cell counts during the first 6 months was so high and completely different from the increment rate experienced after 6 months (Fig. 1), the model was fitted for repeated CD4 count data measured starting from the 6th month. Moreover, the CD4 count data before the initiation of ART (baseline CD4 count) was taken as a covariate.

**Fig. 2:**
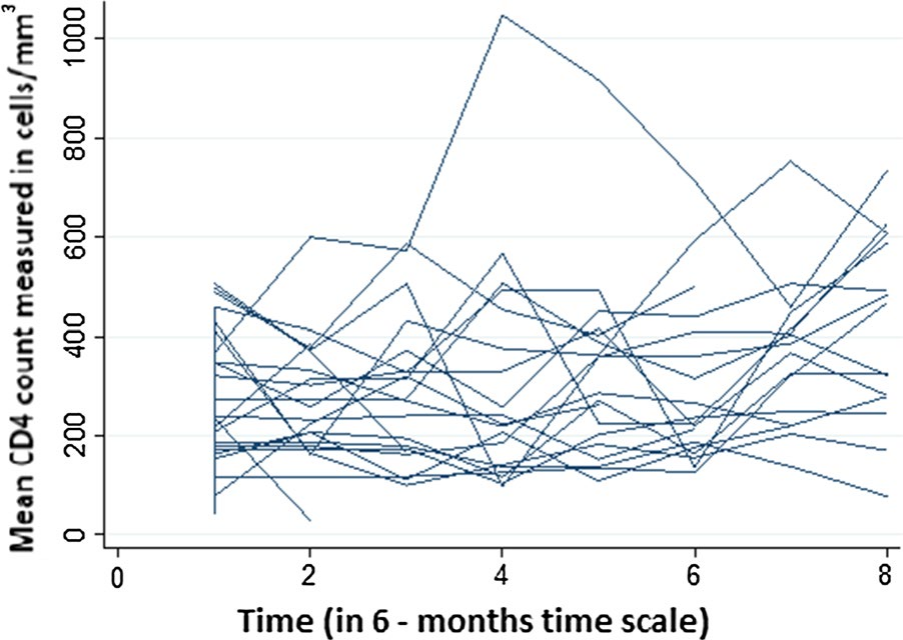
Individual trajectories of the CD4 count of the first 30 ART attendants over time, Felege Hiwot Referral Hospital, 2010–2014

For a more exploratory purpose, individual trajectories were graphed for the first 30 patients CD4 count (Fig. 2). As one could easily understand from the graph, the intercepts of individual trajectories had a range of almost 200 cells/mm^3^ which was a considerably large difference. Similarly, some trajectories were steeper while others were almost horizontal, indicating the possible variability in the slope of CD4 counts. Therefore, because of the variability in the intercept and slope of trajectories, using a mixed model could fit the data very well.

### Modeling the CD4 cell count data

The Intra-class Correlation Coefficient (ICC) which is the variability among intercepts relative to total variability is calculated to be 67.8 % for the null model. This value is greater than the minimum recommended value (25 %) to use a mixed model [17]. The high value of ICC had also further strengthened the appropriateness of using a mixed model for the data. Therefore, the modeling process was started with the null (the unconditional mean) model (Table 2).

**Table 2:** Variability of intercepts of the null model, Felege Hiwot Referral Hospital, 2010–2014

Parameter	Estimate	p value
Residual	21041.196	<0.01
Variance for intercepts	44296.370	<0.001

Next to the null model, the unconditional linear model containing a random intercept and time as both fixed and random effect variable was fitted. In this second model, the fixed intercept and slope were 350.53 and 11.78, respectively, and both were statistically significant (each with a p value of less than 0.001). The random error terms of the intercept and slope were also statistically significant (each with a p value of less than 0.001).

The third model included fixed predictors from different levels to explain the variability among CD4 counts. As a result, three levels, including repeated CD4 count values, individual subjects, and residence were identified as first, second, and third levels of the data structure, respectively. Therefore, time since the initiation of ART (from level-1), baseline age, baseline CD4 count, baseline WHO clinical stage, sex (all from level-2), and average distance between residence and the hospital (from level-3) were considered to see their effect on CD4 count. In line with this, an intercept only random effect was used at the first block to account for the correlation between individuals in the same district or locality. Random intercept and linear effects were also requested at the second block to account for the correlation between repeated CD4 counts of each patient.

Therefore, in addition to the fixed intercept, the fixed effects of baseline CD4 count, initial age, baseline functional status, and time were estimated to be statistically significant. The fixed effect of the interaction between baseline functional status and time was also significant. However, sex, baseline WHO clinical stage, distance from patient’s residence to the hospital, and the interaction of other categorical variables with time were not found to be independent predictors of CD4 count. Before presenting the results of this third model, the significance of its fit was compared with the two models considered so far to facilitate the process of model selection (Table 3).

**Table 3:** The fit and residual values of three models, Felege Hiwot Referral Hospital, 2010–2014

Model	BIC	Residual error
The null model	67978.414	21041.20
Unconditional linear model	67790.95	18744.68
A model included other predictors	67259.324	18600.83

*BIC* bayesian information criteria

Accordingly, because the third model containing other fixed predictors had the smallest BIC value, it was selected as the best fit for the data. For instance, when the null model was compared with the third one, there was a statistically significant difference in fit because the change in BIC = 719.08, and this was significant at Chi square with 9 df (degrees of freedom). The residual error variance also dropped from 21041.20 to 18600.83, implying that about 11.6 % of the within individual variation in CD4 count was associated with the linear effect of independent variables considered. In line with determining the fixed effects, the random effects were also determined by comparing different covariance structures. In this regard, the unstructured covariance structure gave the least information criterion and was selected to model the random effect.

Therefore, it was found that keeping all other variables constant, for a unit increase in the baseline CD4 count, the CD4 count of a participant would increase by 0.820 cells/mm^3^. For each additional half year, the CD4 count of ambulatory or bedridden patients would increase on average by 8.69 cells/mm^3^ (1.45 cells/mm^3^ per month or 17.38 cells/mm^3^ per year) (p value <0.001). However, for working status patients, the mean CD4 count increment for each subsequent half year was 6.59 cells/mm^3^ more when compared to ambulatory or bedridden patients’ or a total gain of 15.28 (8.69 + 6.59) cells/mm^3^ which was equivalent to 2.55 cells/mm^3^ per month or 30.56 cells/mm^3^ per year. The model intercepts of ambulatory/bedridden and working status patients were 377.6 and 358.5 (377.6−19.1), respectively. For each additional 1 year of baseline age, the CD4 count would decrease by 5.0 cells/mm^3^. However, females’ CD4 count was not found to be statistically significantly different from that of males (Table 4).

**Table 4:** Fixed effects on CD4 count, Felege Hiwot Referral Hospital, Ethiopia, 2010–2014

Characteristics	Coefficient	95 % CI		p value
		Lower	Upper	
Intercept	377.6	359.38	455.58	<0.001
Baseline CD4	0.818	0.73	0.91	<0.001
Baseline age	−5.0	−6.0	−3.95	<0.001
Sex of a patient				
Female	17.11	−5.52	39.74	0.238
Male	0.0			
Baseline WHO clinical stage				
WHO stage 1 and 2	−15.33	−48.01	17.35	0.358
WHO stage 3 and 4	0.0			
Time in treatment	8.69	4.34	13.03	<0.001
Functional status (baseline)				
Working	−19.1	−29.1	−9.1	<0.001
Ambulatory/bedridden	0.0			
Functional status × time in treatment				
Working × time in treatment	6.59	1.49	11.68	0.011
Ambulatory/bedridden × time in treatment	0.0			

### The covariance structure

The adequacy of the model fitted could also be affected by the error covariance structure selected. This is because the distribution of errors requires which error covariance structure should be employed. As a result, three different variance- covariance structures were compared for each candidate model. Because the unstructured matrix gave the least information criterion in all possible combinations, it was selected to determine the random effect (Table 5).

**Table 5:** Comparing covariance structures for a model with fixed predictors and random effects, Felege Hiwot Referral Hospital, 2010–2014

Covariance structure	−2LL	AIC	BIC
Unstructured	67225.147	67233.155	67259.324
Compound symmetry	67757.63	67763.636	67788.451
AR (1)	67758.375	67767.636	67803.264

−*2LL* log likelihood, *AIC* akakie information criteria, *BIC* Bayesian information criteria

However, the variance of intercepts at block 1 (a block that measures the variability of parameters from different districts within which individuals were clustered) was not found to be significantly different from zero (Variance = 3167.58, p value = 0.123). Conversely, both random intercept and random slope at block 2 (a block that measures the variability of parameters of repeated data clustered within individuals) were significantly different from zero (Table 6). Specifically, the variance of the random deviations about the fixed intercept and fixed slope of time were 25686.53 and 382.80, respectively, each with a p value of less than 0.001.

**Table 6:** Random effects of intercepts and slopes at block 2 for the a model with fixed predictors, Felege Hiwot Referral Hospital, 2010–2014

Random effect	Estimate	95 % CI for the estimate		p value
		Lower	Upper	
UN (1, 1)	25686.53	22065.43	29901.87	<0.001
UN (1, 2)	−1059.39	−1641.44	−477.33	<0.001
UN (2, 2)	382.80	275.87	531.18	<0.001

*UN* unstructured covariance

## Discussion

In this multi-level analysis to measure the effect of different covariates on the CD4 count over time, the random effect from the third level was not significant (Table 3). This can also be confirmed by the variability of random intercepts (3167.58) which was much less than the residual variability (18600.83). Therefore, the contribution of the variability of CD4 count among individuals clustered by districts at the third level was not found to be statistically significant (block 1).

Similarly, the effect of a fixed variable from the third level which was the average distance of patients’ residence from the hospital was not found to be an independent predictor of CD4 count. However, most studies done in developing countries [18, 19], including a systematic review of studies conducted only in sub-Saharan Africa [20], reported that distance as one component of geographic or transportation-related barriers could end up with poor HIV-related outcomes. Long travel time to the clinic could also force individuals to come late to the service center [21] which could again result in poor health outcome as the immune system could already be damaged [13].

On the contrary, few studies reported favorable impacts of geographic and transportation-related barriers on HIV outcomes [22, 23] among which distance was one of the barriers. As a justification to this paradoxical finding, it was reported that for patients who lost to follow up from a clinic, greater distance to the service center was associated with increased likelihood of re-establishing care at different sites; this was because different clinics were established with the aim of decentralizing the service [22], and this situation could have improved their adherence and then their health outcome [24, 25]. Partly it could also be due to fear of stigma that could force HIV patients to go to distant service centers for their anonymity, which is a common practice in most African settings [26]. Such persons who are highly motivated and intentionally prefer distant health care facilities to minimize stigma could have better HIV treatment adherence [27] that could change the usual association between distance and HIV- related health outcomes into a paradoxical one.

Therefore, if there are possibilities for both positive and negative associations between distance and HIV- treatment outcomes, it is also possible to get a null finding just similar to that of the current study. This is partly because the factors that could give positive and negative outcomes could nullify each other when present almost equivalently in the population under investigation. Other studies, for instance, a study done in Uganda [28] obtained a null finding because the design used excluded participants living more than 20 km from the clinic. Exclusion of participants from long distance could leave the population under investigation nearly homogeneous, regarding distance from the service center.

The other possible reason for the null result in the current study is that even though some individuals’ localities could be closer to the hospital when measured in kilometers, they might not be accessible as there are some rural villages that do not have transportation facilities and could require 4–6 h walk. However, as can be seen from the data, participants who lived in towns 200 km from the service center, for example, could easily access the services because of the availability of transportation infrastructure which could improve their adherence level and probably their treatment outcome [25]. The existence of such contradictory evidence about ART users who live close to and far from services centers could lead to indeterminable results.

However, in the second (individual) level, some of the reasons for the variability of CD4 counts were determined to be baseline CD4 count, baseline age, time since the initiation of ART, baseline functional status, and its interaction with time. Baseline CD4 count was positively associated with CD4 count during treatment; for each subsequent baseline CD4 count, the average gain in CD4 count during the treatment is about 0.818 cells/mm^3^. Some studies also reported positive associations between these characteristics [14]. Other studies reported conflicting evidence claiming a negative association between baseline CD4 count and CD4 count during the treatment period [6, 8]. In fact, the nature of the parameters estimated in these contradicting studies is different. The current study measured just the average gain in CD4 count for each additional baseline CD4 count; but the studies which reported negative associations estimated the average rate of increment or slope per unit time which could diminish over time until it reaches a plateau.

Baseline age was negatively associated with CD4 count response which was supported by other studies [6, 29]. Different authors reasoned out that a favorable influence of younger age on CD4 cell recovery while a patient is receiving HAART may be explained by more-effective thymic function [29]. It was also shown that older persons come to the clinic late [21], and that could place them into poor response to ART treatment [13]. On the contrary, other studies reported a null association between age and CD4 count increment [8, 30]. This could probably be because the majority of the patients were less than 50 years old [8], and 50 % of the participants were between 31 and 41 years old [30] in the respective studies, and in both cases the relative homogeneity in age could dilute its effect on CD4 count increment.

When adjusted for other variables, sex was not found to be an independent predictor of CD4 count which was in agreement with other findings [5, 8]. In contrast, other authors reported that females had better response to ART as compared to males [7]. One common justification is that females could attend voluntary counseling and testing as part of their routine health care services during pregnancy, while male patients are poor in their health seeking behavior as they experience lower rates of HIV testing, repeated-testing, and acceptance of linkage to HIV-care after a positive result [31]. As a result, females are more likely to be diagnosed for infection earlier than males [21, 32], and this could make the response to ART treatment poor for late diagnosed males as the immune system could be damaged irreversibly in advanced stages of the disease [13]. However, early presentation and health seeking behavior might have been indifferent for males and females in the present study population giving a statistically insignificant result.

The two levels of patients’ baseline functional status were found to be statistically significantly different both in their initial status (intercept) and linear change (slope) of their CD4 count. Another study done in eastern Ethiopia also reported a significant association between functional status and change of CD4 count [33]. Opportunistic infections at the start of ART [30] and during the treatment period could affect the CD4 count change over time. Thus, patients who were at a ‘working’ status could be in a better position with regard to co-infection and similar reasons that could influence the response to ART than their counter parts (‘ambulatory/bedridden’).

Time since the initiation of ART was also an independent predictor of CD4 count. Other authors also reported that from 3 months onward, a greater cumulative proportion of time spent with a virus load <400 copies/mL was associated with a more favorable change in CD4 cell count [8]. In the present study, for patients who had ‘working’ functional status, the mean CD4 count would increase by 30.56 cells/mm^3^ per year (p value = 0.011). The corresponding value for ‘ambulatory/bedridden’ patients was even smaller (17.38 cells/mm^3^ per year). These values were smaller than the finding of another study that reported 38.74 % cells/mm^3^ [5]. It was also much less than a study that estimated it at 60 % [8].

Similarly, the gain in CD4 count in the present study was much less than the recommend value which is 50 to 100 cells/mm^3^ per year so that a patient should respond to treatment after 3 months of initiation of ART until a steady state level is reached [4]. Of course, there is some difficulty of comparing it directly with the recommended value because the present study modeled data obtained 6 months after the initiation of the treatment, while the standard is set for data obtained after 3 months of initiation, and the 3 month difference could have brought some difference in the estimated slope [4] as the CD4 count in earlier times is usually high. However, because the observed increment rates were so small compared to the recommended value, it is very difficult to expect the increment to be in the recommended range for similar period of CD4 count data measurement.

In the current study, the 378 participants who continued until the end of year 4 getting the treatment in the hospital had a mean (+SD) CD4 count of 453 (+222) cells/mm^3^ at year 4, which was much less than the CD4 count of healthy HIV negative Ethiopians, reported as 747 (+333) by a study done on Akaki factory workers [34] and 820 (+270) by a study done at the University of Gondar Hospital [35]. This signifies that patients were less likely to reach the local normal range of CD4 count after 4 years of treatment. Moreover, only 37.6 % of them reached a CD4 count of 500 or more cells/mm^3^, while the corresponding values reported by other authors were 45.2 % [30] and 59 % [36]. Of course, partly this could probably be because Ethiopians might have a relatively lower normal CD4 count, compared to most other study populations. For instance, it was lower than that of the Dutch [34], Ugandans [37], and Tanzanians [38]; and if the normal CD4 count is relatively low, the magnitude of increment could also be comparatively smaller. However, as the observed differences are considerably large and all the evidences mentioned are multi-faced, the poor response to ART could be a real problem.

Finally, in this second (individual) level, the variability of initials (intercepts) of the CD4 count due to individual trajectories and the corresponding rates of increment (slopes) were found to be statistically significant (each with p value <0.001). Moreover, the variability between the intercepts (25686.53) was greater than the within variability (18600.83). This indicates that the other reasons for CD4 count variability which was not explained by the fixed variables considered (base line age, baseline CD4 count, time in treatment, and functional status) were represented by the random effects of intercept and slope at individual levels (block 2). This is also the variability captured by the random effect due to uncontrolled (unmeasured) variables.

### Limitations

There are some limitations that should be considered in this paper. Firstly, because the study was based on record review and data was not available for some relevant variables (e.g. patients’ nutritional status), the effect of such unmeasured variables were not estimated separately. However, because a random effect model was used, the contribution of all unmeasured variables was pooled out from the total variability to get the adjusted effect of the independent variables considered. Secondly, data should have been available at least on the 3rd month after the initiation of ART where the increment of CD4 count was high so that the trend will be observed in detail and be comparable with more other studies and standards set for better management.

## Conclusion

In this study, higher baseline CD4 count, younger age, working functional status, and time in treatment contributed positively to the increment of CD4 count. However, the observed increment at 4 year was unsatisfactory as the proportion of ART users who reached the normal range of CD4 count (for the general healthy adult population) was very low. To see their long term treatment outcome, it requires further research with sufficiently longer follow up data. In line with this, the local normal CD4 count should also be investigated for better comparison and proper disease management.

## Abbreviations

ART: antiretroviral therapy
LMM: linear mixed model
GEE: generalized estimation equation
BIC: Bayesian information criteria
ICC: intraclass correlation coefficient
SD: standard deviation
WHO: World Health Organization

## References

[1] Central Statistical Agency, ICF International (2011). Ethiopian demographic and health survey.

[2] Mellors JW, Munoz A, Giorgi JV, Margolick JB, Tassoni CJ, Gupta P (1997). Plasma viral load and CD4+ lymphocytes as prognostic markers of HIV-1 infection. Ann Intern Med.

[3] Panel on Antiretroviral Guidelines for Adults and Adolescents (2011). Guidelines for the use of antiretroviral agents in HIV-1-infected adults and adolescents.

[4] Kaufmann RG, Perrin L, Pantaleo G, Opravil M, Furrer H, Telenti A (2003). CD4 T-lymphocyte recovery in individuals with advanced HIV-1 infection receiving potent antiretroviral therapy for 4 years: the Swiss HIV cohort study. Arch Intern Med.

[5] Montarroyos RU, Miranda-Filho CD, Ce´sar CC, Souza VW, Lacerda RH, Albuquerque MP (2014). Factors related to changes in CD4+ T-Cell counts over time in patients living with HIV/AIDS: a multilevel analysis. PLoS ONE.

[6] Florence E, Lundgren J, Dreezen C, Fischer M, Kirk O, Blaxhult A (2003). Factors associated with a reduced CD4+ lymphocyte count response to HAART despite full viral suppression in the Euro SIDA study. HIV Med..

[7] Addisu A, Dagim A, Tadele E, Adissu A, Mussie A, Filmon K (2015). CD4 cell count trends after commencement of antiretroviral therapy among HIV infected patients in Tigray, northern Ethiopia: a retrospective cross-sectional study. PLoS ONE.

[8] Smith JC, Sabin AC, Youle SM, Loes KS, Lampe CF, Madge S (2004). Factors influencing increases in CD4 cell counts of HIV positive persons receiving long-term highly active antiretroviral therapy. J Infect Dis.

[9] Ebonyi OA, Agbaji OO, Anejo-Okopi AJ, Oguche S, Agaba AP, Sagay SA (2014). Factors associated with a low CD4 count among HIV-1 infected patients at enrolment into HAART in Jos, Nigeria. Br J Med Res.

[10] Bonjour MA, Montagne M, Zambrano M, Molina G, Lippuner C, Wadskier FG (2008). Determinants of late disease-stage presentation at diagnosis of HIV infection in Venezuela: a case-case comparison. AIDS Res Ther.

[11] Louis C, Ivers LC, Smith FM, Freedberg KA, Castro A (2007). Late presentation for HIV care in central Haiti: factors limiting access to care. AIDS Care.

[12] Shastri S, Boregowda HP, Rewari BB, Tanwar S, Shet A, Kumar AMV (2013). Scaling up antiretroviral treatment services in Karnataka, India: impact on CD4 counts of HIV-infected people. PLoS ONE.

[13] Gea-Banacloche J, LH LC (1999). Immune reconstitution in HIV infection. AIDS.

[14] Gandhi TR, Spritzler J, Chan E, Asmuth MD, Rodriguez B, Merigan CT (2006). Effect of baseline- and treatment-related factors on immunologic recovery after initiation of antiretroviral therapy in HIV-1-positive subjects: results from ACTG 384. J Acquir Immune Defic Syndr.

[15] Frehiwot N, Mizan K, Seble M, Fethia K, Tekalign M, Zelalem T (2014). National guidlines for comperhensive HIV prevention, care and treatment.

[16] Singer JD, Willett JB (2003). Applied longitudinal data analysis.

[17] Heinrich JC, Laurence ELJ (2001). Means and ends: a comparative study of empirical methods for investigating governance and performance. J Public Admin Res Theory..

[18] Biadglign S, Derbew A, Amberbir A, Derbie K (2009). Barriers and facilitators to antiretroviral medication adherence among HIV-infected paediatric patients in Ethiopia: a qualitative study. SAHARA J..

[19] Wasti SP, Simkhada P, Randall J, Freeman JV, Van Teijlingen E (2012). Barriers to and facilitators of antiretroviral therapy adherence in Nepal: a qualitative study S. J Health Popul Nutr.

[20] Lankowski JA, Siedner JM, Bangsberg RD, Tsai CA (2014). Impact of geographic and transportation-related barriers on HIV outcomes in Sub-Saharan Africa: a systematic review. AIDS Behav.

[21] Kwobach CM, Braitstein P, Koech JK, Simiyu G, Mwanqi AW, Siika AM et al. Factors associated with late engagement to HIV care in Western Kenya: a cross-sectional study. J Int Assoc Provid AIDS Care. 2015. pii 2325957414567682. 10.1177/2325957414567682 25589304

[22] Geng HE, Glidden VD, Bwana BM, Musinguzi N, Emenyonu N, Muyindike W (2011). Retention in care and connection to care among HIV-infected patients on antiretroviral therapy in Africa: estimation via a sampling-based approach. PLoS One.

[23] Massaquoi M, Zachariah R, Manzi M, Pasulani O, Misindi D, Mwaqomba B (2009). Patient retention and attrition on antiretroviral treatment at district level in rural Malawi. Trans R Soc Trop Med Hyg.

[24] Kimeu M, Burmen B, Audi B, Adega A, Owuor K, Arodi S (2016). The relationship between adherence to clinic appointments and year-one mortality for newly enrolled HIV infected patients at a regional referral hospital in Western Kenya. AIDS Care..

[25] Wood E, Hogg RS, Yip B, Harrigan PR, VO M, O’Shaughness MV (2004). The impact of adherence on CD4 cell count responses among HIV-infected patients. J Acquir Immune Defic Syndr.

[26] Mekonnen Y, Sanders R, Tibebu S, Emmart P (2010). Equity and access to ART in Ethiopia: activity report.

[27] Katz TI, Ryu EA, Onuegbu GA, Psaros C, Weiser DS, Bangsberg RD (2013). Impact of HIV-related stigma on treatment adherence: systematic review and metasynthesis. J Int AIDS Soc..

[28] Haberer EJ, Kiwanuka J, Nansera D, Ragland K, Mellins C, Bangsberg RD (2012). Multiple measures reveal antiretroviral adherence successes and challenges in HIV-infected Ugandan children. PLoS ONE.

[29] Viard PJ, Mocroft A, Chiesi A, Kirk O, Røge B, Panos G (2001). Influence of age on CD4 cell recovery in human immunodeficiency virus-infected patients receiving highly active antiretroviral therapy: evidence from the Euro SIDA study. J Infect Dis.

[30] Kim KH, Yi J, Lee HS (2015). The CD4 slope can be a predictor of immunologic recovery in advanced HIV Patients: a case control study. J Intern Med.

[31] Galdas PM, Cheater F, Marshall P (2005). Men and health help-seeking behaviour: literature review. J Adv Nurs.

[32] Mojumdar K, Vajpayee M, Chauhan N, Mendiratta S (2010). Late presenters to HIV care and treatment, identification of associated risk factors in HIV-1 infected Indian population. BMC Public Health.

[33] Reda AA, Biadgilign S, Deribew A, Gebre B, Deribe K (2013). Predictors of change in CD4 lymphocyte count and weight among HIV infected patients on anti-retroviral treatment in Ethiopia: a retrospective longitudinal study. PLoS ONE.

[34] Aster T, Tsehaynesh M, Tesfaye T, Ermias H, Tefera S, Doorly R (1999). Immunohematological reference ranges for adult Ethiopians. Clin Caccine Immunol..

[35] Addisu G, Biniam M, Beyene M, Meseret W, Lealem G (2014). Establishment of normal reference intervals for CD3+, CD4+, CD8+, and CD4+ to CD8+ ratio of T lymphocytes in HIV negative adults from University of Gondar Hospital, North West Ethiopia.

[36] Kelley FC, Kitchen MRC, Hunt WP, Rodriguez B, Hecht MF, Kitahata M (2009). Incomplete peripheral CD4+ cell count restoration in HIV-infected patients receiving long-term antiretroviral treatment. Clin Infect Dis.

[37] Tugume BS, Piwowar ME, Lutalo T, Mugyenyi NP, Grant MR, Mangeni WF (1995). Haematological reference ranges among healthy Ugandans. Clin Diagn Lab Immunol.

[38] Levin A, Brubaker G, Shao JS, Kumby D, O’Brien TR, Goedert JJ (1996). Determination of T-lymphocyte subsets on site in rural Tanzania: results in HIV-1 infected and non-infected individuals. Int J STD AIDS.

